# Validation of Parkinson's Disease-Related Questionnaires in South Africa

**DOI:** 10.1155/2020/7542138

**Published:** 2020-06-13

**Authors:** Gill Nelson, Ntombizodwa Ndlovu, Nicola Christofides, Tintswalo M. Hlungwani, Irene Faust, Brad A. Racette

**Affiliations:** ^1^School of Public Health, Faculty of Health Sciences, University of the Witwatersrand, 27 St Andrews Rd, Parktown, 2193, South Africa; ^2^UCL Institute for Global Health, Research Department of Infection & Population Health, University College London, London, UK; ^3^Department of Neurology, Washington University School of Medicine, 660 South Euclid Avenue, Campus Box 8111, St. Louis, Missouri 63110, USA

## Abstract

**Background:**

There are very few epidemiological studies investigating Parkinson's disease (PD) in Africa. The hundreds of local languages and dialects make traditional screening and clinical evaluation tools difficult to use.

**Objective:**

The objective of the study was to validate two commonly used PD questionnaires in an African population.

**Methods:**

The PD Screening Questionnaire (PDSQ) and Parkinson's Disease Questionnaire (PDQ-39) were modified and translated into Afrikaans, Setswana, and isiZulu and administered to a sample of healthy local residents. We assessed the internal consistencies and cluster characteristics of the questionnaires, using a Cronbach's alpha test and exploratory factor analysis. The questionnaires were then administered to a population-based sample of 416 research participants. We evaluated the correlations between the questionnaires and both a timed motor task and the Unified Parkinson's Disease Rating Scale motor subsection 3 (UPDRS3), using locally weighted scatterplot smoothing (LOWESS) regression analysis and Spearman's rank correlation.

**Results:**

Both questionnaires had high overall internal consistency (Cronbach's alpha = 0.86 and 0.95, respectively). The modified PDQ-39 had evidence of five subscales, with Factor 1 explaining 57% and Factor 2 explaining 14%, of the variance in responses. The PDSQ and PDQ-39 scores were correlated with the UPDRS3 score (*ρ* = 0.35, *P* < 0.001; and *ρ* = 0.28, *P* < 0.001, respectively).

**Conclusion:**

The translated PDSQ and PDQ-39 questionnaires demonstrated high internal consistency and correlations with clinical severity of parkinsonism and a timed motor task, suggesting that they are valid tools for field-based epidemiological studies.

## 1. Introduction

The global impact of Parkinson's disease (PD) is expected to reach pandemic proportions with the aging of both developed and developing country populations [1–4]. As life expectancy on the African continent increases, neurodegenerative diseases are becoming a growing public health concern. Population-based epidemiological studies of neurodegenerative disease in Africa are rare, and there are limited data from the 21^st^ century [5]. Screening tools designed and validated in the developed world, primarily in English, are not appropriate for much of Africa where there are hundreds of local languages and dialects [6], making screening for PD or parkinsonism using questionnaires validated in countries such as the USA and the UK challenging.

South Africa has the second largest economy in Africa [7] and a predominantly black population with increasing life expectancy [8]. The country has a dynamic academic and healthcare environment and the skills and infrastructure to conduct world-class scholarly activities and provide modern healthcare services. There are 11 official languages, and PD researchers must address this barrier in order to conduct quality Parkinson's disease (PD) research in South Africa. The questionnaires used in developed countries are usually self-administered in English. However, in the South African setting, due to language and literacy challenges, the questionnaires are administered by field workers and are thus subject to misinterpretation when translated. Similarly, some questions do not easily translate from English, and several concepts are not easily translated into African languages.

To ensure that the questions are clear and unambiguous to study participants, it is essential that the English versions be modified, translated into, and validated in vernacular languages where relevant. Failure to do this limits our understanding of disease burden and isolates large populations from the drug development process, in which drugs may perform differently than in predominantly white English-speaking populations in developed countries.

We validated two commonly used PD questionnaires: a PD Screening Questionnaire (PDSQ) [9] and the 39-item questionnaire that assesses self-reported PD-specific health-related quality of life over the last month (PDQ-39) [10], to screen research participants in a field-based epidemiological study of the relationship between environmental manganese (Mn) exposure and parkinsonism. The study population resides near one of the largest Mn smelters in the world. We report the modification and validation of these two questionnaires in English and three of the most commonly spoken languages in South Africa: Setswana, isiZulu and Afrikaans.

## 2. Methods

The Human Research Ethics Committee of the University of the Witwatersrand (ethics clearance certificate no. M141153) and the Human Research Protection Organization of Washington University approved the study.

The PD Screening Questionnaire comprises nine questions to elicit self-reported symptoms typical of PD to screen populations for PD, answered as “yes” or “no.” It has been widely used in epidemiological studies. The PDQ-39 is a validated PD-specific quality of life questionnaire comprised of 39 questions that assess mobility, activities of daily living, emotional well-being, stigma, social support, cognitions, communication, and bodily discomfort in patients with PD. Answers are based on a 5-point Likert scale (never, occasionally, sometimes, often, and always or cannot do at all). The UPDRS3 quantifies motor features of PD on a 0–108 scale with higher scores indicating more severe parkinsonism.

We used a three-step validation process to validate the two questionnaires: translation and face validity, content validity, and construct validity.

In the first step, we modified and translated several questions in the English versions of the questionnaires, in accordance with methods described by Aaronson et al. [11]. The questions were modified without changing the information they attempt to elicit, in order to make them understandable to English-speaking South Africans, in terms of both language and content. The modified questionnaires were reviewed for accuracy and appropriateness, after which individuals proficient in three of the most common South African languages (Setswana, isiZulu, and Afrikaans) then translated them. The questionnaires were back-translated by different individuals, also proficient in the respective languages.

In the second step (content validation), we tested the performance of the translated questionnaires in 16 English-, Afrikaans-, Setswana-/Sotho-, and isiXhosa-/isiZulu-speaking male and female volunteers with varying educational achievements. Setswana and Sotho are linguistically similar and have similar origins, as have isiXhosa and isiZulu which are both Nguni languages. Interviewers who were fluent in the relevant languages were trained by skilled qualitative researchers to administer the questionnaires, face to face. The interviewers recorded participants' responses to each question and asked them to clarify their thought processes in order to elicit what they understood by each question. Probing was used to obtain a detailed narrative of the cognitive process of each participant before he/she answered a question. The interviews were audio-recorded, transcribed verbatim, and translated into English. Based on the results of these cognitive interviews, minor changes were made to the questionnaires. For example, in almost all South African vernacular languages, there is no single word for “balance”. Thus, in the PDSQ, the question “Is your balance poor?” was changed to “Is your balance poor when walking?”. The two questionnaires were then administered to a convenience sample of 160 individuals, older than 40 years, from a residential area in the Midvaal region of South Africa, who spoke one of the four languages (40 per language) to assess the internal consistency of the questionnaires and to determine if the PDSQ and PDQ-39 retained their item cluster characteristics.

In the third step (construct validity), we administered the questionnaires, face to face, to 416 participants in a population-based study of the health effects of environmental Mn exposure. All participants were older than 40 years and were local residents, living near a ferromanganese smelter. Local, trained fieldworkers recruited study participants, using a random household sampling method. Performances on the PDQ-39 and PDSQ were validated against two methods of assessing motor function: (1) a Purdue Grooved Pegboard timed test [12] was used to measure fine motor speed and visuomotor coordination (times taken to complete the task with the dominant and nondominant hand were recorded), and (2) the Unified Parkinson's Disease Rating Scale motor subsection 3 (UPDRS3) [13] was administered by a movement disorders specialist without knowledge of the responses in either questionnaire. The tests and tools are described in Supplementary Table S1.

### 2.1. Statistical Analysis

We performed all statistical analyses using Stata MP version 14.2 (StataCorp LP, College Station, Texas). The internal consistencies of both the PDSQ and the PDQ-39 were assessed using a Cronbach's alpha test; internal consistency was considered acceptable when *α* > 70, based on the 160 completed questionnaires. For the PDQ-39, we calculated the internal consistency of the eight subscales in the original questionnaire. To determine if the two questionnaires retained their original subscale structures (one for PDSQ and eight for PDQ-39), we used exploratory factor analysis with an orthogonal rotation (Varimax) to explore how many factors the items loaded onto the modified and translated versions of the questionnaires. Exploratory factor analysis provides procedures for determining an appropriate number of factors and the patterns of loadings from the data [14]. We used an eigenvalue >1.0 as the threshold for a domain (i.e., subscale) [15–17]. We then recalculated the internal consistency of the five subscales that were identified through the exploratory factor analysis.

To investigate the association between UPDRS3 scores and age, grooved pegboard times (dominant and nondominant hand), PDQ-39 score, and PDSQ score, we first graphically evaluated these associations using locally weighted scatterplot smoothing (LOWESS) regression analysis. We also compared the performance of the PDSQ in relation to the PDQ-39. We again graphically evaluated this association using LOWESS regression analysis and evaluated correlations using Spearman's rank correlation, with significance at *α* = 0.05.

## 3. Results

The demographic characteristics of the study participants in the content and construct validity steps are described in Table 1. In both the content and construct validity components, the majority of study participants were female (61.5% and 58.9%, respectively). The mean ages of the two study populations were 50.9 ± 10.0 (range 33–79) years and 51.2 ± 9.4 (range 38–97) years, respectively.

The mean UPDRS3 score of those who participated in the construct validity component (*N* = 416) was 10.1 ± 7.8 (Table 2). The range of scores associated with the PDSQ and the PDQ-39 was wide (0–52). Participants with higher UPDRS3 scores were older (*ρ* = 0.30, *P* < 0.001) and took longer to complete the grooved pegboard test (dominant hand *ρ* = 0.39, *P* < 0.001; nondominant hand *ρ* = 0.39, *P* < 0.001).

The mean PDSQ score was 0.63 ± 1.85 for the English-speaking participants, 0.73 ± 1.47 for the Afrikaans-speaking participants, 2.48 ± 2.99 for the Setswana-speaking participants, and 3.15 ± 3.47 for the isiZulu-speaking participants. The differences were statistically significant (*F* = 8.41, *P* < 0.001). Factor analysis for the modified PDSQ demonstrated loading on one factor (eigenvalue 3.81), consistent with assessing a single clinical domain. The PDSQ had high overall internal consistency (Cronbach's alpha = 0.86); the individual questions also demonstrated high internal consistency (Cronbach's alpha range = 0.83–0.86), suggesting that a participant's response to one question was related to his/her response to other questions in the PDSQ (Table 3).

The PDQ-39 scores differed by language: 57.25 ± 17.82 for the English, 55.41 ± 18.19 for the Afrikaans, 63.76 ± 17.64 for the Setswana, and 70.95 ± 33.41 for the isiZulu group (*F* = 2.84; *P* = 0.04). Factor analysis demonstrated that, instead of the eight subscales in the original PDQ-39, the modified PDQ-39 had five subscales, with Factor 1 (eigenvalue = 14.08) explaining 65% of the variance in responses, Factor 2 explaining 15% (eigenvalue = 3.30), Factor 3 explaining 9% (eigenvalue = 1.94), Factor 4 explaining 6% (eigenvalue = 1.42), and Factor 5 explaining 6% (eigenvalue = 1.21) across all participants. The five subscales comprised (1) mobility, out-of-house, and activities of daily living; (2) emotional well-being; (3) cognitive-communication-body discomfort; (4) activities of daily living inside the house (ADL); and (5) items relating to eating and drinking in public, problems in relationships, and unintentionally falling asleep (Table 4). The modified and translated PDQ-39 questionnaire demonstrated high overall internal consistency (Cronbach's alpha = 0.95). However, the individual subscales had variable internal consistency (Table 5). Subscales for mobility (*α* = 0.9), activities of daily living (*α* = 0.82), emotional well-being (*α* = 0.9), and body discomfort (*α* = 0.83) demonstrated good to excellent internal consistency. Social support (*α* = 0.78), cognition (*α* = 0.79), and stigma (*α* = 0.74) had acceptable internal consistency. Communication (*α* = 0.69) demonstrated marginal internal consistency.

**Figure 1:**
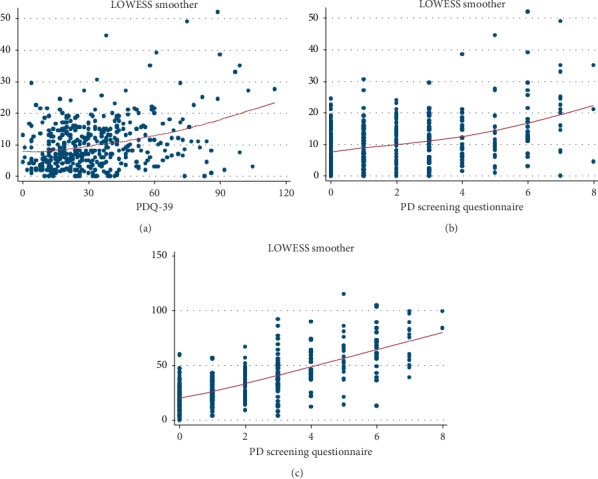
Association between (a) severity of parkinsonism and PDQ-39 score (*ρ* = 0.22); (b) severity of parkinsonism and PDSQ score (*ρ* = 0.30); (c) PDQ-39 and PDSQ scores (*ρ* = 0.64).

The UPDRS3 scores were positively correlated with both the PDSQ (*ρ* = 0.30; *P* < 0.001) and the PDQ-39 (*ρ* = 0.22; *P* < 0.001) scores, indicating that, in general, those with more severe parkinsonism had more PD symptoms and poorer PD-specific health-related quality of life (Figure 1). The PDQ-39 and PDSQ scores were strongly correlated (*ρ* = 0.64; *P* < 0.001) (Figure 1).

## 4. Discussion

In this multistep validation study, we demonstrated that the two questionnaires (the PDSQ and the PDQ-39) retained their original meanings and had good internal consistency once modified and translated for the most common South African languages. Moreover, we demonstrated strong correlations between severity of parkinsonism and PD symptoms as well as PD-specific health-related quality of life, suggesting that the questionnaires performed as expected when compared to a clinical measure of severity. The PDSQ was originally designed for use in epidemiological studies to identify people with PD, due to the high cost of this expert assessment in large populations [18]. While the purpose of the population-based study focuses on identifying parkinsonism in an environmentally exposed population, the PDSQ performed as expected, given that the number of affirmative answers was positively associated with the UPDRS3 score. The modified PDQ-39 also demonstrated good internal consistency and was strongly correlated with motor signs of parkinsonism in the population-based study (the PD-specific questions were omitted). Overall, we provided evidence that these two modified questionnaires are valid screening tools for use in field-based epidemiological studies and may augment data obtained from a clinical specialist's examination.

The use of the PDSQ questionnaire in the population-based study is largely consistent with the original, intended use of this tool as a screening questionnaire in epidemiological studies. However, we used parkinsonism as a continuous measure of a health outcome, as opposed to a diagnosis of PD. This approach is better suited to investigating associations between motor signs of parkinsonism and environmental exposures in population-based studies of adults. A recently published study in Xhosa-speaking South Africans found that only 18% of the participants could correctly identify a patient with PD from a video [19]. Although we focused on Setswana- and isiZulu-speaking South Africans, PD awareness in these groups is likely to be similar. Nevertheless, the symptoms in the modified PDSQ appeared to be recognized by the participants, and those with higher scores on the PDSQ tended to also have higher UPDRS3 scores. Further research is needed to determine if these modified African language questionnaires have sufficient sensitivity and specificity to be used as screening questionnaires to identify those with PD. However, in this environmental health research setting, the questionnaires performed well.

While our study is not the first to attempt to validate the PDQ-39 in non-English languages, our aim to determine the association between an environmental exposure- and PD-specific quality of life is unique. We previously demonstrated higher scores on the PDQ-39 in those with occupational Mn exposure and UPDRS3 ≥ 15 [20]. In this environmental Mn exposure study, we found similar associations between the modified PDQ-39 and severity of parkinsonism, demonstrating a consistency across different Mn-exposed populations. While the modified PDQ-39 retained the anticipated association with parkinsonism, the clustering of questions (subscales) differed from the original British version of the questionnaire [10]. Interestingly, a validation study in which the PDQ-39 was modified from British English to American English also changed the questionnaire item cluster characteristics, finding that mobility-stigma-cognition, ADL-communication-body discomfort, and well-being-social support clustered together [21]. Overall, the agreement between the subcategories of the PDQ-39 was excellent, but social support, stigma, and communication were outliers, potentially due to ethnic differences in our study population. For example, questions categorized as stigma and social support in the British and American versions of the questionnaire had a very different context for the black South African research participants. Older black South Africans often live with extended families and might not need support from a spouse/partner, which might influence the relationships between questions. The stigma questions were not asked unless the study participant said that he/she had a diagnosis of PD; this might have impacted the agreement with the rest of the questionnaire. In addition, very few study participants indicated that they had been diagnosed with PD, which could also have reduced the internal reliability. The questions about stigma might not be essential in population-based epidemiological studies. The reason for communication demonstrating poorer agreement with the other variables is less clear. The use of the word “people” in two of the three questions in this subcategory might be too broad. Communication entails interacting beyond the realm of friends and family. English and Afrikaans are spoken by more than half of the larger community in the study area (approximately 54%) [22], most of whom are white, and are the primary languages used in the workplace. Many of the study participants spoke neither language. The obstacle of language, when communicating with the general public, is not uncommon for those with low education levels and for those educated in schools where English is not the language of instruction.

Despite the overall good performance of the modified questionnaires in this South African population-based study, the study had some limitations. The internal consistency of the PDSQ differed across the four languages, and more adaptations might be required before it is used in population-based studies. For example, there might be overreporting of symptoms (e.g., smaller handwriting) due to low literacy rather than neurological problems. The PDQ-39 scores were lower for English and Afrikaans speakers, which could be an indication of literacy rather than quality of life; English and Afrikaans speakers might be better educated and more literate than those who are not fluent in either language.

The use of the PDQ-39 in this screening context is nontraditional and beyond the original intent of the questionnaire. However, the strong correlation with a clinically meaningful motor examination from a movement disorders specialist provides compelling evidence that the questionnaires are measuring PD-specific effects on quality of life. While other validation studies of these questionnaires were performed in general population samples, there is also substantial evidence that the PDQ-39 is a valid measure of the impact of PD on quality of life in patients with PD [23–25]. The fact that our study participants were not PD patients might explain some of the differences between our study findings and previous reports.

In summary, our study provides strong evidence that the modified PDQ-39 is suitable for use in epidemiological studies investigating parkinsonism in English-, Afrikaans-, Setswana-, and isiZulu-speaking South African communities, while the PDSQ might need to be further modified before it can be used. Further validation in PD patients whose primary languages are Afrikaans, Setswana, and isiZulu will be necessary before the questionnaires can be recommended for use in clinical practice.

## Figures and Tables

**Table 1:** Sociodemographic characteristics of participants.

Characteristic	Content validity step participants, *N* = 160		Construct validity step participants, *N* = 416	
	*n*	%	*n*	%
Sex				
Male	60	38.5	171	41.1
Female	96	61.5	245	58.9

Education				
None	2	1.4	59	14.8
Primary school	15	10.4	98	24.6
Secondary school	96	66.7	229	57.4
Tertiary	31	21.5	13	3.3

Home language				
English	40	26.0	1	0.3
Afrikaans	37	24.0	4	1.1
Sotho	46	29.9	234	62.6
isiXhosa	9	5.8	55	14.7
Setswana	4	2.6	14	3.7
isiZulu	18	11.7	66	17.7

Ever smoked				
Yes	44	28.9	147	35.3
No	108	71.1	269	64.7
Ever consumed alcohol regularly				

Yes	53	34.2	226	54.3
No	102	65.8	190	45.7

**Table 2:** Measurement scores obtained by participants (construct validity step).

Measurement	*n*	Mean	SD	Min	Max
UPDRS3	416	10.1	7.8	0	52
Dominant hand Grooved Pegboard time	414	106.7	44.7	43.2	300
Nondominant hand Grooved Pegboard time	408	116.9	47.7	51.4	300
PDQ-39	412	33.1	21.3	0	115
PDSQ	416	1.9	2.1	0	8

UPDRS3, Unified Parkinson's Disease Rating Scale motor subsection 3; PDQ-39, Parkinson's Disease Questionnaire; PDSQ, Parkinson's Disease Screening Questionnaire; SD, standard deviation.

**Table 3:** Internal reliability (Cronbach's alpha) of the modified PDSQ.

Question	*n*	Alpha
Do you have trouble rising from a chair?	160	0.85
Is your handwriting smaller than it once was?	160	0.85
Do people tell you that your voice is too soft?	159	0.86
Is your balance poor?	158	0.84
Do your feet suddenly seem to get stuck when turning or in doorways?	160	0.83
Do people tell you that you have a blank look on your face?	158	0.85
Do your arms or legs shake?	156	0.84
Do you have trouble doing up your buttons?	157	0.85
Do you shuffle your feet or take tiny steps when you walk?	159	0.83
Overall Cronbach's alpha		0.86

PDSQ, Parkinson's Disease Screening Questionnaire.

**Table 4:** Internal reliability (Cronbach's alpha) of the modified PDQ-39.

Subscale/item	*n*	Alpha
Mobility		0.90
Had difficulty doing the leisure activities you would like to do?	160	0.95
Had difficulty looking after your home, e.g. repairs, housework, cooking?	159	0.95
Had difficulty carrying bags of shopping?	160	0.95
Had problems walking to the shops or church?	157	0.95
Had problems walking to your nearest neighbor?	159	0.95
Had problems getting around the house as easily as you would like?	160	0.95
Had difficulty getting around the community?	160	0.95
Needed someone else to help you when you leave the house?	160	0.95
Felt frightened or worried about falling over in public?	158	0.95
Been stuck in the house more than you would like?	160	0.95

Activities of daily living		0.82
Had difficulty washing yourself?	159	0.95
Had difficulty dressing yourself?	160	0.95
Had problems doing up your shoelaces?	160	0.95
Had problems writing clearly?	160	0.95
Had difficulty cutting up food?	158	0.95
Had difficulty holding a drink without spilling it?	160	0.95

Emotional well-being		0.90
Felt depressed?	160	0.95
Felt isolated and lonely?	158	0.95
Felt weepy or tearful?	158	0.95
Felt angry or bitter?	157	0.95
Felt anxious?	157	0.95
Felt worried about your future?	159	0.95

Stigma		0.74
Avoided eating or drinking in public?	158	0.95
Felt worried by other people's reaction to you?	160	0.95

Social support		0.78
Had problems with your close personal relationships?	160	0.95
Lacked support in the ways you need from your spouse or partner?	157	0.95
Lacked support in the ways you need from your family or close friends?	148	0.95

Cognition		0.79
Unintentionally fallen asleep during the day?	147	0.95
Had problems concentrating, e.g. when reading or watching TV?	157	0.95
Felt your memory was bad?	158	0.95
Had distressing dreams or hallucinations?	159	0.95

Communication		0.69
Had difficulty with your speech?	160	0.95
Felt unable to communicate with people properly?	156	0.95
Felt ignored by people?	157	0.95

Bodily discomfort		0.83
Had painful muscle cramps or spasms?	159	0.95
Had aches and pains in your joints or body?	158	0.95
Felt unpleasantly hot or cold regardless of the weather?	159	0.95

PDQ-39, Parkinson's Disease Questionnaire.

**Table 5:** Factor loadings for the five subscales of the modified PDQ-39.

Subscale/items	Factor 1	Factor 2	Factor 3	Factor 4	Factor 5	Uniqueness
Mobility						
Had difficulty doing the leisure activities you would like to do?				0.5068		0.4312
Had difficulty looking after your home, e.g. repairs, housework, cooking?				0.6701		0.4060
Had difficulty carrying bags of shopping?				0.6524		0.3564
Had problems walking to the shops or church?				0.7854		0.2754
Had problems walking to your nearest neighbor?	0.6493					0.3560
Had problems getting around the house as easily as you would like?	0.5024			0.5076		0.4437
Had difficulty getting around the community?						0.4831
Needed someone else to help you when you leave the house?	0.6774					0.4535
Felt frightened or worried about falling over in public?	0.7081					0.3084
Been stuck in the house more than you would like?	0.6207					0.3828

Activities of daily living						
Had difficulty washing yourself?	0.8342					0.2364
Had difficulty dressing yourself?	0.8414					0.2301
Had problems doing up your shoelaces?	0.7047					0.3311
Had problems writing clearly?						0.8624
Had difficulty cutting up food?	0.5278					0.5812
Had difficulty holding a drink without spilling it?	0.6658					0.4081

Emotional well-being						
Felt depressed?			0.5982			0.4076
Felt isolated and lonely?			0.7209			0.2780
Felt weepy or tearful?			0.7222			0.3093
Felt angry or bitter?			0.6391			0.3011
Felt anxious?			0.7564			0.2631
Felt worried about your future?						0.4039

Stigma						
Avoided eating or drinking in public?					0.6980	0.3548
Felt worried by other people's reaction to you?			0.5338			0.3381

Social support						
Had problems with your close personal relationships?					0.5065	0.3413
Lacked support in the ways you need from your spouse or partner?		0.5497				0.5650
Lacked support in the ways you need from your family or close friends?		0.5557				0.4769

Cognition						
Unintentionally fallen asleep during the day?					0.5013	0.4569
Had problems concentrating, e.g. when reading or watching TV?		0.6104				0.4447
Felt your memory was bad?		0.7196				0.4351
Had distressing dreams or hallucinations?		0.5846				0.5028

Communication						
Had difficulty with your speech?		0.6261				0.4952
Felt unable to communicate with people properly?		0.6193				0.4364
Felt ignored by people?						0.6290

Bodily discomfort						
Had painful muscle cramps or spasms?		0.6063				0.4294
Had aches and pains in your joints or body?		0.5619				0.3637
Felt unpleasantly hot or cold regardless of the weather?		0.6999				0.3729

PDQ-39, Parkinson's Disease Questionnaire.

## Data Availability

The data are available, on request, from the corresponding author.

## Abbreviations

PD: Parkinson's disease
PDSQ: Parkinson's Disease Screening Questionnaire
PDQ-39: 39-item Parkinson's Disease Questionnaire
UPDRS3: Unified Parkinson's Disease Rating Scale motor subsection 3
LOWESS: Locally weighted scatterplot smoothing.

## Supplementary Materials

Supplementary Materials Supplementary Table: description and scoring of tests and tools used.
