# Supplementary material for: Validation of Parkinson's Disease-Related Questionnaires in South Africa
Source: Parkinsons Dis. 2020 Jun 13;2020:7542138. doi: 10.1155/2020/7542138 (PMC7306845; doi:10.1155/2020/7542138)
Supplement: Supplementary Materials — Supplementary Table: description and scoring of tests and tools used. [file 7542138.f1.docx]

SUPPLEMENTARY TABLE: Description and scoring of tests and tools used

| **Test/tool** | **Description** | **Scoring** |
| --- | --- | --- |
| PD symptom questionnaire | Comprises nine questions to elicit self-reported symptoms typical of PD to screen populations for PD | Questions are answered as ‘yes’ or ‘no’ |
| PDQ39 | Extensively validated PD-specific quality of life questionnaire comprised of 39 questions to assess mobility, activities of daily living, emotional well-being, stigma, social support, cognitions, communication and bodily discomfort in patients with PD. | Answers are based on a 5-point Likert scale (never, occasionally, sometimes, often, and always or cannot do at all. |
| UPDRS3 | Used to clinically evaluate 14 aspects of motor function | Scores range from 0 - 108 with higher scores indicating more severe parkinsonism |
| Purdue grooved pegboard test | Measures fine motor speed and visuomotor coordination | Times taken to complete the task with the dominant and non-dominant hand are recorded |
